# Substance Use and Associated Factors among University Students in Ethiopia: A Cross-Sectional Study

**DOI:** 10.1155/2014/969837

**Published:** 2014-04-28

**Authors:** Gezahegn Tesfaye, Andualem Derese, Mitiku Teshome Hambisa

**Affiliations:** Department of Public Health, College of Health and Medical Sciences, Haramaya University, P.O. Box 235, Harar, Ethiopia

## Abstract

Studies indicate that substance use among Ethiopian adolescents is considerably rising; in particular college and university students are the most at risk of substance use. The aim of the study was to assess substance use and associated factors among university students. A cross-sectional survey was carried out among 1040 Haramaya University students using self-administered structured questionnaire. Multistage sampling technique was used to select students. Descriptive statistics, bivariate, and multivariate analysis were done. About two-thirds (62.4%) of the participants used at least one substance. The most commonly used substance was alcohol (50.2%). Being male had strong association with substance use (AOR (95% CI), 3.11 (2.20, 4.40)). The odds of substance use behaviour is higher among third year students (AOR (95% CI), 1.48 (1.01, 2.16)). Being a follower of Muslim (AOR (95% CI), 0.62 (0.44, 0.87)) and Protestant (AOR (95% CI), 0.25 (0.17, 0.36)) religions was shown to be protective of substance use. Married (AOR (95% CI), 1.92 (1.12, 3.30)) and depressed (AOR (95% CI), 3.30 (2.31, 4.72)) students were more likely to use substances than others. The magnitude of substance use was high. This demands special attention, emergency preventive measures, and targeted information, education and communication activity.

## 1. Introduction

The use of substances such as alcohol, khat, and tobacco has become one of the rising major public health and socioeconomic problems worldwide [1]. The global burden of disease attributable to alcohol and illicit drug accounts 5.4% of the total burden of disease. Another 3.7% of the global burden of disease is attributable to tobacco use. And disorders due to psychoactive substance use including alcohol, drug, and tobacco dependence are the main underlying conditions ultimately responsible for the largest proportion of the global burden of disease attributable to substance use [2].

The rapid economic, social, and cultural transitions that most countries in sub-Saharan Africa are now experiencing have created a favorable condition for increased and socially disruptive use of drugs and alcohol [3]. Substance misuse is a growing problem in Ethiopia, as in many developing countries. Alcohol and khat are the most frequent substances of abuse [4]. According to the Ethiopian Demographic and Health Survey (DHS) 2011, the prevalence of alcohol use among men and women is 53% and 45%, respectively, and 11% of women and 28% of men ever chewed khat [5].

Studies indicate that substance use among Ethiopian adolescents is considerably rising [6, 7]. Of the young segment of the Ethiopian population, college and university students are at the highest risk of substance use. Joining university often leads to new opportunities, independence from family control, self-decision making, and peer-pressures to use or abuse alcohol or other drugs [8].

Among some studies done in Ethiopian universities and colleges, a study in Axum University showed a lifetime prevalence of khat chewing 28.7%, alcohol drinking 34.5%, and cigarette smoking 9.5% [9]. A study in Debre Markos, Northern Ethiopia, found the life time prevalence of substance use to be 14.1% [10]. A study done among college students in Northwest Ethiopia revealed a lifetime prevalence of 13.1% for cigarette smoking and 26.7% for khat chewing [6]. A study in Addis Ababa University showed that 31.4% ever drunk alcohol, 14.1% ever chewed khat, 8.7% ever smoked cigarette [8]. A study in Jimma University showed prevalence of khat chewing, cigarette smoking, and alcohol intake to be 33.1%, 21.3%, and 36.4%, respectively [11].

Even if substance use has become a common problem among university students in Ethiopia, only scant information is available about the magnitude of substance use and factors contributing for its use in this segment of the population. Furthermore, the effect of mental health status of students such as depression on substance use was not well explored. Therefore the aim of this study was to assess the magnitude of substance use and associated factors among university students in Ethiopia.

## 2. Methods

### 2.1. Study Area

The study was conducted from April 15–30, 2013 in Haramaya University, which is located 510 Km away from Addis Ababa in the East Hararghe Zone between Harar and Dire Dawa towns. It is one of the oldest Universities next to Addis Ababa University in the country. There are three campuses in the University (main campus, Harar campus, and Chiro campus). The study included main campus and Harar campus. During the time of the study, the University has 12 Colleges and 55 departments with 15,183 regular students in undergraduate study.

### 2.2. Study Design

Institution based cross-sectional study was conducted.

### 2.3. Source Population

All undergraduate students at Haramaya University were the source population for the study.

### 2.4. Study Population

All students who were randomly selected by multistage sampling from source population. Those regular undergraduate students, who are not blind and not critically sick (to the extent of being unable to read and write) during the time of data collection, were included.

### 2.5. Sample Size Determination

The sample size (*n* = 1040) was primarily determined for the study of prevalence and factors associated with depression, suicidal ideation and attempt among Haramaya University students. It was determined by taking the prevalence of suicidal ideation among Zambian university students (31.9%) [12], 95% confidence level, marginal error of 4%, and 10% non-response rate. Using single population proportion formula, the sample size becomes 520. Since the sampling was multistage, design effect of 2 was taken and the final total sample size becomes 1040.

### 2.6. Sampling Procedure

**Figure 1:**
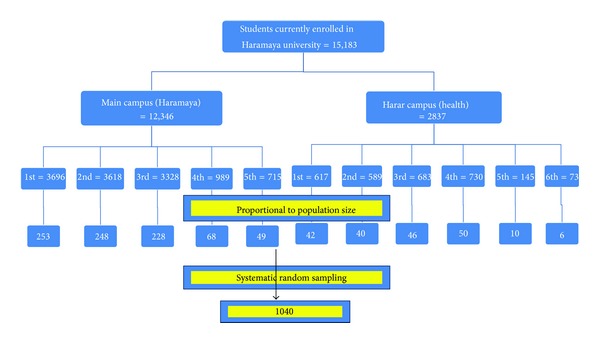
The schematic presentation of the sampling procedure employed to select participants in Haramaya University, April 2013.

Multistage sampling technique was used to select the study participants. First, students were divided into two by campuses (Main campus and Harar Campus). Then, further stratification was done based on the year of study. Finally, systematic random sampling technique was applied to select students in each year of study from the list of students' name in their respective batch. Students from each year of study were allocated proportionally to their class size (Figure 1).

### 2.7. Data Collection Procedure and Tools

The questionnaire was developed after extensive review of literatures and similar study tools used previously by adapting to the purpose of the study. The data collectors were master's degree holders who have guided the students to complete the questionnaire. The data collectors explain each question to the students to help them understand the questions well and fill their own response on questionnaire. Facilitators were academic staffs who were familiar with the specific college who facilitated the smooth running of data collection process before and during data collection period. The principal investigators have followed and controlled overall data collection process, trained data collectors and facilitators, and performed pretest. The data was collected using self-administered structured questionnaire which was prepared in English and then translated to local language (Amharic) which most students could understand.

### 2.8. Study Variables

*Independent Variables.* Sociodemographic characteristics (age, sex, religion, marital status, monthly pocket money), year of study, depression status, and type of high school attended.

*Dependent Variable.* The dependent variable is substance use.

### 2.9. Data Analysis

Descriptive statistics was done to describe the study population. Bivariate and multivariate analysis were employed to identify factors associated with the outcome variable. Odds ratio with 95% confidence interval was computed to assess the level of association and statistical significance. Those variables which were found to be significant in the bivariate analysis were retained for further multivariate analysis. Then logistic regression analysis was done to control confounding variables and to predict independent factors associated with substance use.

### 2.10. Data Quality Assurance

The questionnaire was pretested on students of the nearby Dire Dawa University. The data collectors (facilitators) were trained, and proper instruction was given before the survey. The collected data were reviewed and checked for completeness before data entry.

### 2.11. Operational Definition

*Substance Use.* In this study it was referred to as use of at least one of the substances (alcohol, khat, cigarettes, and illicit drugs) in an individual's life time to alter mood or behaviour.

*Current User.* A person who consumed any substance at least once in the past 30 days.

*Ever Use.* Referred to as use of any of the substances at least once in an indiviual's life time.

*Illicit Drugs.* It was defined as the use of psychoactive substances such as hashish, cannabis, and heroin, for which the production, sale, or use is prohibited.

## 3. Results

### 3.1. Sociodemographic Characteristics

Out of the total 1040 students that participated in the survey, questionnaires from 1022 respondents were complete and considered for analysis making the response rate 98.3%. Of the total 1022 respondents, 828 (81%) were age ranged from 20 to 24 years with a mean age of 20.9 (SD = ± 2.17 years). From the total participants, 777 (76.0%) were males, 420 (41.1%) were Oromos by ethnicity, 518 (50.7%) were Orthodox Christians, 928 (90.8%) were never married, 352 (34.4%) were first year students, 703 (68.8%) were originally from urban area, 874 (85.5%) have attended public high school, and 430 (42.1%) get a monthly pocket money of 300–499 Ethiopian birr (Table 1).

### 3.2. Magnitude of Substance Use

Among the participants, 638 (62.4%) used at least one substance in their lifetime. The study revealed that 419 (41.0%) of the students chewed khat at least once in their lifetime and the current use of khat is 241 (23.6%). Concerning alcohol drinking habits, 513 (50.2%) reported that they drank alcohol at least once in their lifetime while 204 (20%) were drinking alcohol over the last 30 days prior to the study. The study showed that 225 (22%) of the respondents smoked cigarettes at least once in their life time whereas 110 (10.8%) of the respondents have smoked cigarettes in the past 30 days. Furthermore, 178 (17.4%) of the study participants used illicit drugs like hashish at least once in their lifetime. Seventy-six (7.4%) of the participants have used illicit drugs in the last 30 days (Table 2).

### 3.3. Pattern of Substance Use

The respondents were further asked about their chewing pattern. The response indicated that 171 (71%) chew khat sometimes and 70 (29%) of them claimed chewing khat always. Regarding the pattern of drinking, 135 (66.2%) drink sometimes and 69 (33.8%) drink always. Among the current smokers, 107 (81.1%) smoke sometimes and the rest 25 (18.9%) smoke always. Regarding the frequency of using illicit drugs, from those who use illicit drugs currently 79 (44.4%) use sometimes and 15 (55.6%) use always.

### 3.4. Time to Start Substance Use

Out of those who have chewed khat at least once in their life time, the majority 288 (68.7%) started to chew khat before joining university and the rest 131 (31.3%) after joining university. Majority of those who drank alcohol 407 (79.3%) started to drink before joining university and the rest 106 (20.7%) after joining university. More than half of the students 118 (52.5%) who smoked cigarettes once in their life time started to smoke before joining university. About 102 (57.3%) started to use illicit drugs before joining university and 76 (42.7%) started to use illicit drugs after joining university.

### 3.5. Reasons for Substance Use

Different reasons were mentioned by students for the use of drugs. The reasons mentioned for chewing khat by the respondents were as follows: 190 (45.4%) to increase academic performance, 108 (25.8%) to get personal pleasure, 92 (23%) to get relief from tension, 68 (16.2%) to stay awake, 68 (16.2%) due to peer influence, 43 (10.3%) due to academic dissatisfaction, 27 (6.4%) to get acceptance by others, 23 (5.5%) to be sociable, 17 (4.1%) to increase pleasure during sex, and 11 (2.6%) due to religious practice.

Among those who reported to drink alcohol, 237 (46.2%) used alcohol to get personal pleasure, 125 (24.4%) to get relief from tension, 89 (17.3%) due to peer influence, 50 (9.8%) to stay awake, 48 (9.4%) to be sociable, 43 (8.4%) to increase pleasure during sex, 25 (4.9%) to increase academic performance, 19 (3.7%) due to academic dissatisfaction, 12 (2.3%) to get acceptance by others, and 5 (0.9%) due to religious practice.

The reasons mentioned for smoking cigarettes were as follows: 88 (39.1%) to get relief from tension, 63 (28%) to increase academic performance, 45 (20%) to stay awake, 42 (18.7%) due to peer pressure, 29 (12.9%) to get personal pleasure, 21 (9.4%) to be sociable, 19 (8.5%) due to academic dissatisfaction, 14 (6.3%) to increase pleasure during sex, 12 (5.4%) to get acceptance by others, and 5 (2.3%) because of religious practice.

There are many reasons mentioned by students for use of illicit drugs: 88 (49.4%) to get relief from tension, 50 (28.1%) to increase academic performance, 42 (23.6%) to get personal pleasure, 38 (21.3%) due to peer influence, 32 (18%) to stay awake, 15 (8.4%) due to academic dissatisfaction, 14 (7.9%) to get acceptance by others, 14 (7.9%) to be sociable, 11 (6.2%) due to religious practice, and 9 (5.1%) to increase pleasure during sex.

### 3.6. Factors Associated with Substance Use

Initially different variables such as age, sex, amount of pocket money, background, year of study, religion, marital status, type of high school attended, and depression status were considered for bivariate analysis. In the bivariate analysis the following variables showed a statistically significant association with substance use: sex, year of study, religion, marital status, type of high school attended, and depression. These variables were taken and analyzed together using multivariate logistic regression model.

After controlling for the effects of potentially confounding variables using multivariate logistic regression model, sex, marital status, year of study, religion, and depression were found to be statistically significant predictors of substance use. Being male had strong association with substance use (AOR (95% CI), 3.11 (2.20, 4.39)). The odds of substance use are higher among third year students (AOR (95% CI), 1.48 (1.01, 2.16)) than others. There was no statistically significant association between fourth year and above students and substance use. Being a follower of Muslim (AOR (95% CI), 0.62 (0.44, 0.87)) and Protestant (AOR (95% CI), 0.25 (0.17, 0.36)) religions were shown to have less odds of having substance use. Those students who were ever married (AOR (95% CI), 1.92 (1.12, 3.30)) were more likely to use substance than never married. The odds of substance use were three times higher among students who have depression compared to those who do not have depression (AOR (95% CI), 3.30 (2.31, 4.72)) (Table 3).

## 4. Discussion

In this study the overall prevalence of substance use for at least one substance was 62.4%. The most commonly used substances in descending order were alcohol (50.2%) khat (41%), cigarettes (22%), and other illicit drugs (17.4%). Sex, marital status, year of study, religion, and depression were found to be statistically significant predictors of substance use.

The overall prevalence of substance use for at least one substance was 62.4%. This is slightly lower than a similar study in Kenyan universities which was 69.8% [13] but higher than a study in Axum University, Northern Ethiopia, where the life time prevalence of substance use was 45.9% [9]. The difference in magnitude from that of Axum University might be due to the difference in the study area where in this part of the country there is easy availability and accessibility of substances especially khat and alcohol which are frequently taken by students, and are relatively socially acceptable due to different sociocultural environment.

The lifetime prevalence of khat chewing was 41%. This result is higher than the result of study done among high school students in Eastern Ethiopia 24.3% [14], a study done in college students in North West Ethiopia 26.7% [6], and in Jazan district of Saudi Arabia 21.4% [15]. This may be because the eastern part of the country where the study area is located is the major khat producer for domestic consumption and foreign export, so that students would have easy access to khat with cheap costs. The main reasons given by the study participants for chewing khat were to increase academic performance, to get personal pleasure, to get relief from tension, and to stay awake. This is in line with other research done in Jimma and Butajira [16, 17].

The prevalence of lifetime alcohol use in this study was 50.2%, which is similar to a study in Kenya 51.9% [13] and is much higher than Addis Ababa University medical students 31.4% [8]. The difference from the Addis Ababa University may be due to the fact that the study was entirely conducted by involving only medical students in contrast to ours in which we have selected participants from all categories of students.

In the present study the life time prevalence of cigarette smoking was 22% which is higher than a study in Axum University 9.3% [9] and a study in Saudi University students 14.5% [18] but lower than a study in Kenya 42.8% [13] and a study in Medical University of Białystok 30.8% [19]. The higher magnitude as compared to Axum and Saudi University may be due to the high use of khat among students in our study than those studies, as there is relationship between khat chewing and cigarette smoking. The variation in the result with Kenya and Białystok might be because of the differences in study setting.

The life time prevalence of illicit drug use in this particular study is 17.4%, which is the least substance used by the students. It is apparent from this finding that relatively few students had tried illicit drugs compared to other substances. This might be due to the fact that students did not get these illicit drugs easily, and the possession and use of these drugs result in penalty under the law of the country. Furthermore this could be attributed to either underreporting due to self-reporting or a lack of availability of these substances. Nevertheless, this finding appears consistent with previous studies [13, 20, 21] where reports of illicit drug use have been low.

The study found out that being male had strong association with substance use (AOR (95% CI), 3.11 (2.20, 4.39)). The reason could be due to the fact that in male students the level of substance exposure is high and peer pressure is more common than female students. Moreover, many of the substances such as khat, tobacco, and alcohol are socially acceptable if practiced among males. A similar study done in Debremarkos Poly Technique College, Northern Ethiopia [10], found similar results in which substances abuse in males was three and half times higher than in female respondents.

Those third year students (AOR (95% CI), 1.48 (1.01, 2.16)) were more likely to use substances than others. The reason may be due to the fact that students in third year were at middle of their campus life in which they usually become hopeless and get into depression so that they may tend to use substances to get relieved from the depression mood. This result was not supported with previous studies [8, 9] in which year of study has no association with use of any of the specific substances.

In contrast to a study in Axum University, Northern Ethiopia [9], in this study being a follower of Muslim (AOR (95% CI), 0.62 (0.44, 0.87)) and Protestant (AOR (95% CI), 0.25 (0.17, 0.36)) religions were shown to have less odds of having substance use as compared to Orthodox Christians. The difference with Muslim religion might be due to the fact that Muslims commonly use khat than other substances which hide the overall substance use in this population as they do not usually use other substances.

Married students (AOR (95% CI), 1.92 (1.12, 3.30)) were more likely to use substance than never married. This was not consistent with another study [22] in which marriage was associated with reduced risk of substance use disorders. The difference may be due to the difference in study subjects in that the study used sample of subjects selected from households in the community in contrast to the current study which entirely used university students. The disparity in the result may be due to the fact that, unlike married people in the general population, married university students may suffer the effect of marital condition and own family departure on their day to day campus life that may lead them to use substances more than single students who were relatively at a low stress level.

The odds of substance use were three times higher with students who have depression compared to those who do not have (AOR (95% CI), 3.30 (2.31, 4.72)). This result is similar to a study in Turkey [23] in which substance use risk was found to be higher in those with higher depression scores. This could be due to the fact that depressed students are more prone to use substances to relief themselves from the stress or depression mood.

The study has strength in that it involved students from all categories of field of study and used larger sample size. Moreover, the study attempted to see the presence of any association between depression status of students and substance use behaviour. The study has limitations such as restriction of the study to only undergraduate students and used self-administered questionnaire which needs students to give self-reported use of substances that may tend to underestimate substance use.

## 5. Conclusions

The overall life time prevalence of substance use among university students is high. The most commonly used substance among students is alcohol. Sex, marital status, year of study, religion, and depression were found to be independent predictors of substance use among students. Substance use among university students demands special attention, emergency preventive measures, and targeted IEC activity. Education and awareness creation on harmful effect of substance use especially among male students should be done. There should be a unit in the university that is responsible for counselling of depressed patients and strategies should be designed to prevent depression among university students.

## Figures and Tables

**Table 1:** Sociodemographic characteristics of students in Haramaya University, April 2013.

Variables	Frequency (*n* = 1022)	Percent
Sex		
Male	777	76.0
Female	245	24.0
Age group in years		
≤19	165	16.1
20–24	828	81.0
≥25	29	2.8
Ethnicity		
Oromo	420	41.1
Amhara	304	29.7
Guraghe	81	7.9
Tigre	67	6.6
Wolayita	29	2.8
Somali	15	1.5
Others*	106	10.4
Religion		
Orthodox	518	50.7
Muslim	245	24.0
Protestant	214	20.9
Others**	45	4.4
Marital status		
Never married	928	90.8
Ever married	55	39
Year of study		
1st year	352	34.4
2nd year	265	25.9
3rd year	249	24.4
4th year and above	156	15.3
Place of residence before		
Urban	703	68.8
Rural	319	31.2
Type of high school attended		
Public high school	874	85.5
Nonpublic high school	148	14.5
Monthly pocket money (Ethiopian Birr)		
≤100	221	21.6
101–299	240	23.5
300–499	430	42.1
500–999	105	10.3
≥1000	26	2.5

*Other ethnic groups include Harari, Sidama, and Hadiya; **other faiths (Catholic, Jehovah Witness, and Traditional).

**Table 2:** Life time and current use of different substances by sex among students in Hramaya University, April 2013.

Substances	Sex		Total (%)
	Male (%)	Female (%)	
Any substance			
Yes	524 (67.4)	114 (46.5)	638 (62.4)
No	253 (32.6)	131 (53.5)	384 (37.6)
Ever use of khat			
Yes	370 (47.6)	49 (20.0)	419 (41.0)
No	407 (52.4)	196 (80.0)	603 (59.0)
Current use of khat			
Yes	223 (28.7)	18 (7.3)	241 (23.6)
No	554 (71.3)	227 (92.7)	781 (76.4)
Ever use of alcohol			
Yes	418 (53.8)	95 (38.8)	513 (50.2)
No	359 (46.2)	150 (41.2)	509 (49.8)
Current use of alcohol			
Yes	179 (23.1)	25 (10.2)	204 (20.0)
No	598 (76.9)	220 (89.8)	818 (80.0)
Ever use of cigarette			
Yes	197 (25.4)	28 (11.4)	225 (22.0)
No	580 (74.6)	217 (88.6)	797 (88.0)
Current use of cigarette			
Yes	101 (13.0)	9 (3.8)	110 (10.8)
No	676 (87.0)	236 (96.2)	912 (89.2)
Ever use of illicit drugs			
Yes	160 (20.6)	18 (7.3)	178 (17.4)
No	617 (79.4)	227 (92.7)	844 (82.6)
Current use of illicit drugs			
Yes	72 (9.3)	4 (1.6)	76 (7.4)
No	705 (90.7)	241 (98.4)	946 (92.6)

**Table 3:** Factors associated with substance use at least once in life time among students in Haramaya University, April 2013.

Variables	Substance use		COR, 95% CI	AOR, 95% CI	*P* value
	Yes	No			
Sex					
Male	524	253	**2.38 (1.78, 3.19)**	**3.11 (2.20, 4.39)**	**<0.001**
Female	114	131	1	1	
Religion					
Orthodox	364	154	1		
Muslim	154	91	**0.72 (0.52, 0.99)**	**0.62 (0.44, 0.87)**	**0.06**
Protestant	86	128	**0.28 (0.20, 0.40)**	**0.25 (0.17, 0.36)**	**<0.001**
Others*	34	11	1.31 (0.65, 2.65)	0.93 (0.44, 1.99)	0.860
Marital status					
Never married	567	361	1		
Ever married	71	23	**1.97 (1.21, 3.20)**	**1.92 (1.12, 3.30)**	**0.017**
Year of study					
1st year	213	139	1		
2nd year	157	108	0.95 (0.69, 1.31)	0.83 (0.58, 1.19)	0.307
3rd year	175	74	**1.54 (1.09, 2.18)**	**1.48 (1.01, 2.16)**	**0.045**
4th year and above	93	63	0.96 (0.66, 1.42)	1.22 (0.79, 1.88)	0.365
High school					
Public high school	565	309	1		
Nonpublic high school	73	75	**0.53 (0.38, 0.76)**	0.76 (0.50, 1.14)	0.178
Depression					
Nondepressed	417	331	1		
Depressed	221	53	**3.31 (2.37, 4.62)**	**3.30 (2.31, 4.72)**	**<0.001**

*Others faiths include Catholic, Jehovah Witness, and traditional.
